# Ultra-Thin, Short-Focus, and High-Aperture Metalens for Generating and Detecting Laser Optical Vortices

**DOI:** 10.3390/nano12152602

**Published:** 2022-07-28

**Authors:** Anton Nalimov, Victor Kotlyar

**Affiliations:** 1 IPSI RAS—Branch of the FSRC “Crystallography and Photonics” RAS, Molodogvardeyskaya 151, 443001 Samara, Russia; kotlyar@ipsiras.ru; 2 Technical Cybernetics Department, Samara National Research University, Moskovskoye Shosse 34, 443086 Samara, Russia

**Keywords:** topological charge, optical vortex, multifocal metalens

## Abstract

A combined high-aperture metalens in a thin silicon nitride film that consists of two tilted sectored metalenses is considered. Each sector of the metalens consists of a set of binary subwavelength gratings. The diameter of the metalens is 14 μm. Using a time-domain finite difference method, we show that the metalens can simultaneously detect optical vortices with two topological charges −1 and −2, almost over the entire spectrum of visible wavelengths. The metalens can distinguish several wavelengths that are focused at different points in the focal plane due to a 1-nm change in wavelength resulting in a focal spot shift of about 4 nm. When the metalens is illuminated by a Gaussian beam with left-handed circular polarization, two optical vortices with topological charges 1 and 2 are simultaneously formed 6-μm apart at the focal distance of 6 μm.

## 1. Introduction

Over the past years, combined metalenses have been investigated intensively [1,2,3,4]. Researchers have proposed various variants of multifocal lenses [1,2], as well as combined metasurfaces matched to each other [3,4]. There are different ways to create metasurfaces [5]. Most often, metalenses are designed in the form of pillars of different shapes that act as resonators for a given wavelength and polarization [6,7,8,9,10,11,12]. At the same time, if not using materials with a high refractive index, these pillars need to have a high aspect ratio and the accuracy of transverse dimensions needs to be very high, which increases the cost of their manufacture. Broadband metalenses are also known to focus coherent light, which may be polarized both linearly [13] and circularly [14].

One of the applications of metalenses or metasurfaces is the collimation of light for coupling light into/out of a waveguide [15]. If the waveguide is multimode and the modes are separated by wavelengths, then a problem of wavelength-division multiplexing of the output light has to be solved. For example, diffraction gratings are well known to do this [16]. However, the shortcoming of this approach is the need to use two separate devices—a lens for focusing light and a diffraction grating. Another interesting application of metalenses is for generating optical vortices with a topological charge [7,17]. Utilizing the topological charge (TC), additional information can be encoded in the beam, which increases the amount of the information transferred by the optical fiber. Metalenses can also be used to detect the topological charge [18,19,20] or polarization of laser beams [21]. For example, authors in [21] proposed a metalens that can focus light into different circles depending on the type of polarization of incident light. In [22], a combined metalens was considered, which focuses three longitudinal vortices with distinct topological charges at different focal planes on the optical axis.

In this paper, we describe a combined metalens capable of collimating the light, detecting a TC in a beam, and wavelength-division multiplexing of the beams. Using such the metalens, up to three wavelengths can be separated simultaneously, as well as different TC. The proposed metalens can be used in mode-division multiplexing devices in information transmission lines via optical fibers. The advantages are that the lens is broadband and can combine all three devices in one. Therefore, the alignment of the device is simplified due to a reduced number of optical components.

## 2. Theoretical Background

A method for designing metalenses in a thin amorphous silicon film for the visible light spectrum was described in [23]. Briefly, it is as follows. In diffractive optics, a spiral zone plate (SZP) is known to focus an optical vortex [24]. The transmission function of the binary phase SZP has the form

(1) $$T_{m}(r,\varphi )=\mathrm{sgn}\left[\cos\left(m\varphi +\frac{kr^{2}}{2f}\right)\right],$$

where *m* is the topological charge (TC) of the optical vortex, (*r, φ*) are polar coordinates, *k* is the wavenumber of light with wavelength λ, *f* is the focal length of the parabolic lens.

The depth of the binary relief of such a phase SZP should be equal to

(2) $$H=\lambda {\left[2(Ren-1)\right]}^{-1},$$

where Re is the real part of the refractive index *n* of the SZP material. The operation of half-wave gratings is based on different effective refractive indices of the grating for two directions of the polarization vector: along the grating lines (TE) and across them (TM) [25]:

$$n_{eff}^{TE}={\left(Qn_{r}^{2}+\left(1-Q\right)n_{m}^{2}\right)}^{1/2},$$

(3) $$n_{eff}^{TM}={\left(Qn_{r}^{-2}+(1-Q)n_{m}^{-2}\right)}^{-1/2},$$

where *Q* is the fill factor (the ratio of the step width to the grating period), *n_r_* is the refractive index of the grating material, and *n_m_* is the index of the medium.

The design of the metalens based on a spiral phase plate for the incident circularly polarized light was considered in detail in [23]. In this work, we design an SZP and a corresponding metalens that can generate two optical vortices with topological charges 1 and 2. This metalens is also shown to detect similar optical vortices but with the negative TC, −1 and −2. The metalens combines two zone plates of orders of 0 and 1. For example, the metalens can be described by a rotation matrix $\hat{R}(\varphi )=\left(\begin{array}{l} \;\;\cos\varphi \;-\sin\varphi \\ -\sin\varphi \;-\cos\varphi \end{array}\right)$ as it rotates the polarization vector by the angle *φ* multiplied by the transmission $e^{im\varphi }$ of the spiral plate and the transmission function $\exp\left(-ikr^{2}/(2f)\right)$ of the spherical lens of the 1st order. Then, when illuminated by the light with left-handed circular polarization (LHCP), the metalens generates an output optical vortex converging into an intensity ring with a topological charge of 2 and right-handed circular polarization (RHCP) [26]:

(4) $$\begin{array}{l} \exp\left(i\varphi -i\frac{kr^{2}}{2f}\right)\left(\begin{array}{l} \;\;\cos\varphi \;-\sin\varphi \\ -\sin\varphi \;-\cos\varphi \end{array}\right)\left(\begin{array}{l} 1\\ -i \end{array}\right)\\ =\exp\left(2i\varphi -i\frac{kr^{2}}{2f}\right)\left(\begin{array}{l} 1\\ i \end{array}\right). \end{array}$$

That is, the metalens constructed using a zone plate of the 0th order creates a 1st order vortex at the focus. Meanwhile, the 1st order zone plate integrated into the (spiral) metalens increases the order by one, so the resulting order equals *m* = 2. If a laser beam with LHCP that has a vortex with TC *m* = −2 falls onto the same metalens, then a focal spot with RHCP and without a vortex will be formed. Hence, we infer that the metalens will focus this vortex into a circular focal spot. Similarly, a metalens intended for detecting the TC of the incident vortex *m* = −1 can be constructed. To do this, the metalens should be created on the basis of a zero-order zone plate (a regular zone plate). This metalens transforms LHCP light into a RHCP vortex with the TC *m* = 1. If the incident light has the TC *m* = −1 and LHCP, then it is transformed to non-vortex RHCP light (*m* = 0) and focused at the focal plane into a round focal spot.

## 3. Simulation of TC Detection

**Figure 1:**
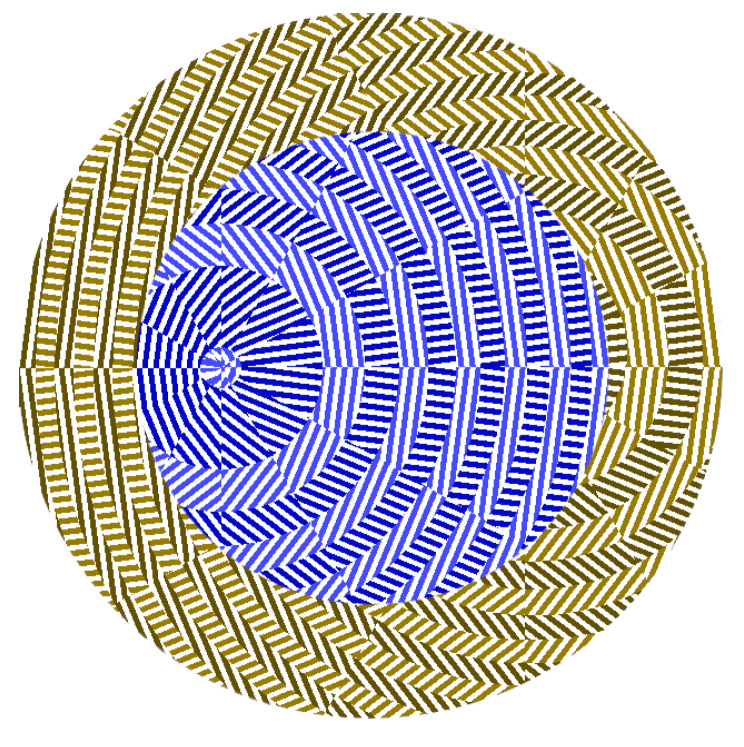
Metalens for detecting an LHCP laser beam with TC *m* = −1 and *m* = −2: white is a metalens material, colored patterns—through grooves. Blue/cyan colors correspond to the inner metalens intended to focus a beam with TC *m* = −1 at point (−3 μm; 0), brown/yellow—for the outer one for a beam with *m* = −2 and a focal point at (3 µm; 0). In the gratings, the darker-colored zones (blue, brown) are rotated by 90 degrees relative to the lighter-colored zones (yellow, cyan) as the corresponding phases in these zones are shifted by π, thus making it possible to focus the incident light. These zones are circular in the inner metalens (cyan/blue areas are round) and spiral in the outer one (brown/yellow). Each zone is divided into 12 sectors, with the direction of gratings in each sector being constant.

If several possible topological charges in a beam need to be detected, then the light should be transmitted through several metalenses. One way is to use a beam splitter and different metalenses, but it is possible to create a combined metalens consisting of several ones, each of which transmits the light to a focal spot with definite coordinates. Shown in Figure 1 is a metalens intended to detect TC *m* = −1 and *m* = −2 in an incident beam with LHCP.

**Figure 2:**
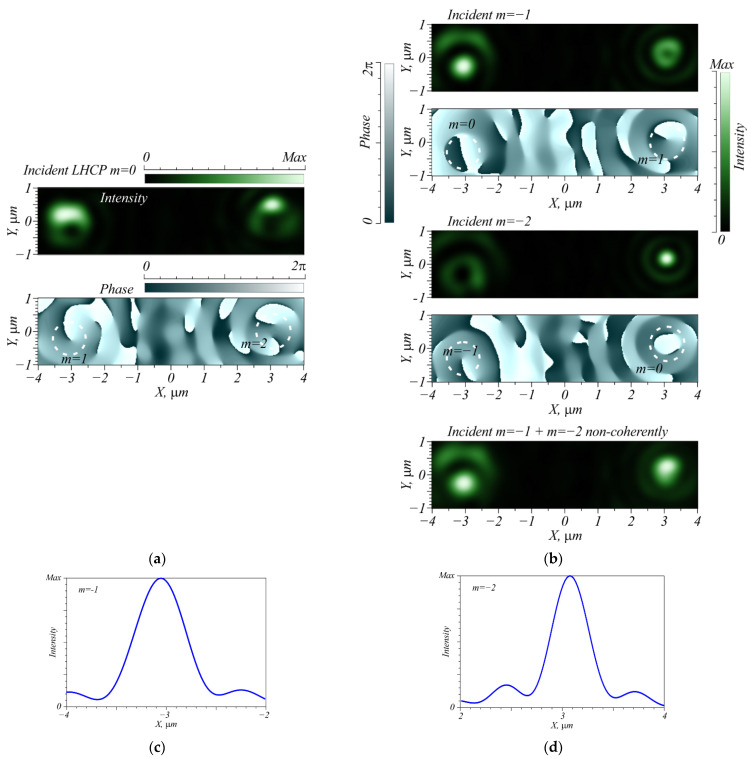
(**a**) Intensity and phase of the output light when the incident light is a non-vortex LHCP plane wave, (**b**) intensity and phase at the metalens output from an incident beam with TC *m* = −1 (**top**), *m* = −2 (**middle**), both TCs at once (**bottom**), and (**c**,**d**) their profiles along the *X* axis. The wavelength is λ = 0.633 µm. Dashed rings imposed on the phase patterns show the location of the vortices.

The first metalens is located inside a circle with an outer radius of 4.9 µm and the second one is within a ring limited by radii 4.9 μm and 7 μm, with the two of them forming a conventional metalens (Figure 1). The parameters are as follows: wavelength is λ = 633 nm, the diameter is 14 µm, the material is SiN (refractive index *n* = 2.04), the microrelief height is 1 µm, the grating period is 220 nm, the focal length and output plane distance is 6 µm (the average numerical aperture is 0.62 for the inner metalens and 0.75 for the outer one). The metalens is intended to focus an incident laser beam with TC *m* = −1 into a focal spot centered at (−3 µm; 0) and a beam with TC *m* = −2 to a focal spot centered at (3 µm; 0). The numerical simulation was carried out using the FDTD method in the FullWave software. Shown in Figure 2 is the operation of the lens. If the incident beam is a LHCP plane wave, then optical vortices are generated at both above-said focal spot coordinates with topological charges *m* = 1 and *m* = 2 (Figure 2a). Figure 2b–d shows the light intensity in the focal plane at the given wavelength for three different incident beams with TC *m* = −1 and *m* = −2.

The laser beam with TC *m* = −1 is seen to form an intensity maximum found close to the calculated coordinates (−3.05 μm; −0.26 μm). Some asymmetry is explained by the fact that when acting as a polarizer, the diffraction gratings have different transmission coefficients depending on the incidence angle [27]. Thus, on the right, an intensity ring is observed near the point (3 μm; 0), since it is the point where the vortex with RHCP and order *m* = 1 is focused. If the initial TC is changed to −2, the round focal spot “moves” to the right, forming an intensity maximum at coordinates (3.08 μm; 0.17 μm). On the left, a ring is formed with a minimum of intensity in its center, since at this point the vortex with RHCP and TC *m* = −1 is focused. Thus, we infer that by analyzing the intensity maximum, the TC of the incident beam can be measured. Since the metalens part which focuses the incident field with TC *m* = −2 is located on the outer ring, its numerical aperture is higher, producing a narrower focal spot (Figure 2d). Note that an inclination of the plane wave in the focus in case of incident vortex with TC *m* = −1 is seen from Figure 2b (where *m* = 0) to be slightly greater than in case of incident beam with *m* = −2. This can be explained by the fact that the beam comes to the point x = −3 µm from the inner part of the metalens, which is located inside a circle with an outer radius of 4.9 µm. At the point x = 3 µm, the light comes to the focus more symmetrically from the outer part of the metalens and with less inclination. If, however, a non-vortex LHCP wave is sent to the metalens, then intensity rings of different diameters are formed at both points. Thus, in the left focus, a vortex with RHCP and TC *m* = 1 is found, with a vortex with RHCP and order *m* = 2—found in the right focus.

## 4. Numerical Simulation of Metalens-Aided Focusing of Different Incident Wavelengths

**Figure 3:**
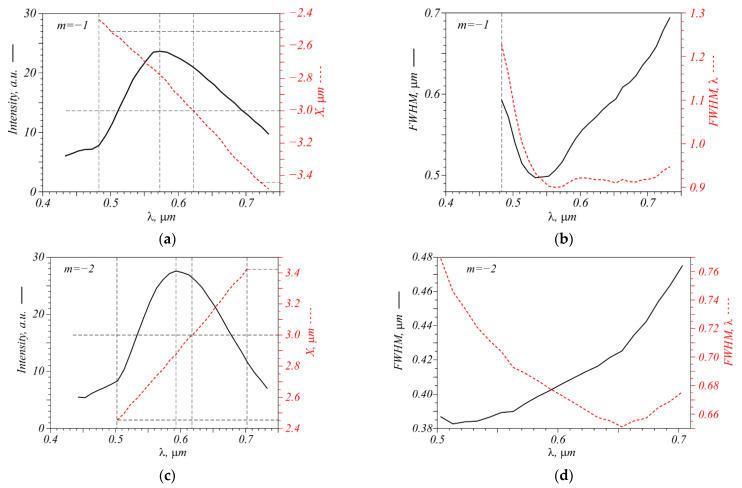
Dependences of the intensity, the X coordinate of the intensity maximum (**a**,**c**), and its width (**b**,**d**) on the light wavelength for the incident field with TC *m* = −1 and *m* = −2.

Mode-division multiplexing in a waveguide can be achieved using multiple wavelengths in one fiber. Therefore, it would be interesting to analyze how the metalens works with different wavelengths. Shown in Figure 3 are plots of the intensity maximum and its X coordinates depending on the incident wavelength, as well as plots of the full width half maximum (FWHM) of the peak for the incident field with TC *m* = −1 and *m* = −2.

The metalens is seen from Figure 3 to operate in a wide range of wavelengths. The TC *m* = −1 can be detected in a beam in the range of wavelengths from 490 to 720 nm. Furthermore, note that if the wavelength of light changes, the focal spot shifts along the *X* axis. In the range from 500 nm to 720 nm, the shift along the *X* axis is approximately 910 nm, while the FWHM of the focal spot does not exceed 600 nm. Therefore, the simultaneous detection of the TC and the wavelength is possible, since with the parameters obtained, it is possible to resolve two maxima at different wavelengths. As a result, it is not necessary to separate the light by wavelengths, which reduces possible losses when detecting the TC. Note that despite the calculated position of the focal spot X = −3 µm at the wavelength λ = 633 nm, it is slightly shifted: the numerical simulation gives a value of X = −3.05 µm. The maximum intensity is obtained at the wavelength of 570 nm.

The same can be said of the beam with TC *m* = −2. In this case, the simultaneous detection of the TC and the wavelength is possible too. The operating range of the metalens is also somewhat shifted towards shorter wavelengths—from 500 to 700 nm, while the range of the focal spot shift along the *X* axis at these wavelengths is 860 nm. The FWHM of the focal spot does not exceed 480 μm, which helps to detect the wavelength using the shift of the intensity maximum. The smallest FWHM for *m* = −1 is approximately 485 nm, for *m* = −2–380 nm. A tendency towards a decrease in the focal spot width when the numerical aperture increases is also seen from Figure 2.

**Figure 4:**
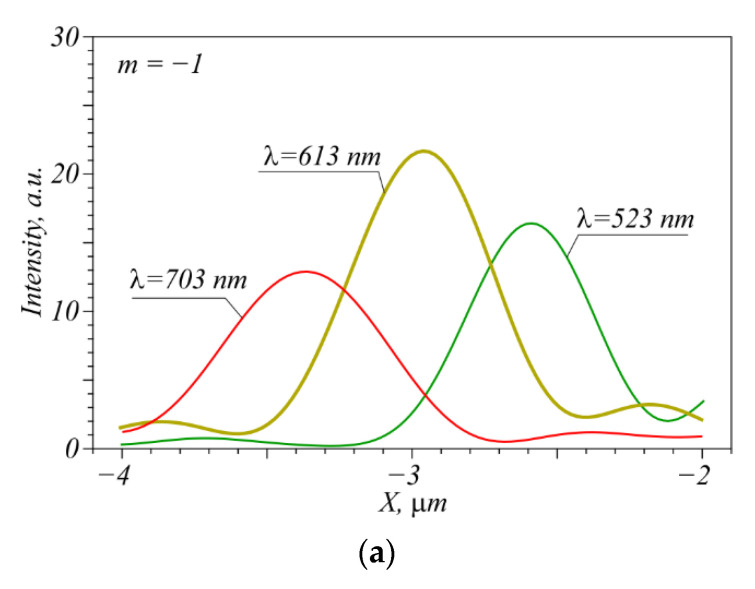

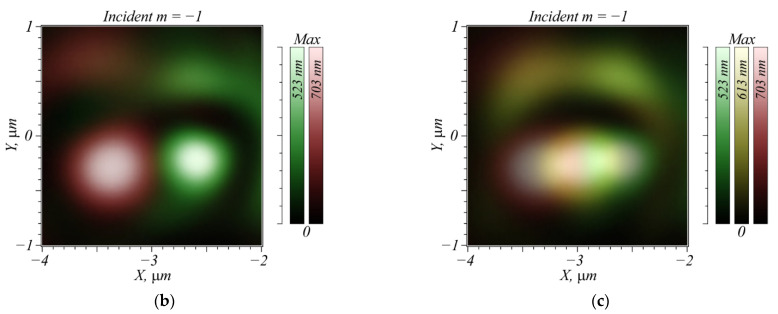
The intensity maxima profiles of a focused vortex with TC *m* = −1 for three incident wavelengths λ = 523 nm, 613 nm, and 703 nm (**a**), simultaneous location of two (**b**) and three (**c**) focused vortices in the focal plane, in accordance with their relative intensities.

Shown in Figure 4 are intensity maximum profiles and focal spot patterns in color for two and three incident wavelengths. The simulation for each wavelength was also performed using a FDTD method, before summing up the intensities with no regard for the light phase, so coherence between the light sources is disregarded. The initial intensities for all three wavelengths are considered to be the same. From Figure 4a,b, the wavelengths of 523 and 703 nm are seen to be resolved irrespective of the presence of both vortices in the incident beam, because the focal spots do not overlap. The signal-to-noise ratio (hereinafter referred to as *p* = *S*/*N*, where *S* is the useful intensity and *N* is the spurious intensity component) is at least 18:1, that is, *p* = 18, where the noise is side lobes around the maximum of the adjacent wavelength (Figure 4a). If an extra vortex of wavelength 613 nm is added while two previous vortices are retained, it can be resolved with *p* = 2.8. The vortex with λ = 613 nm reduces the quality of detection of the vortices at λ = 523 and 703 nm because of its side lobes; however, putting the intensity sensors for these two focal spots on their edges (left and right in Figure 4) and, thus, capturing them partly, without a central peak, can significantly improve the signal-to-noise ratio, which will lead to a successful resolution of all three wavelengths for any combination of them in the incident beam.

**Figure 5:**
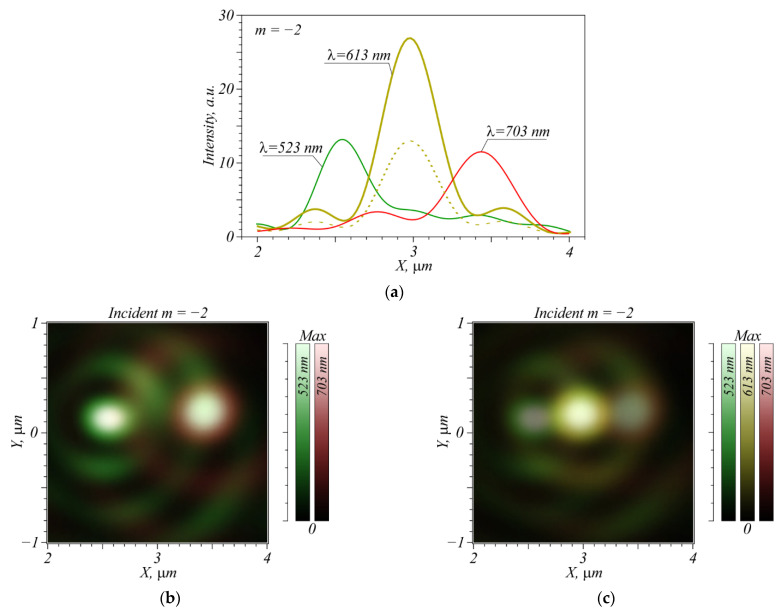
Profiles of intensity maxima of a focused vortex with TC *m* = −2 for three wavelengths: λ = 523 nm, 613 nm, and 703 nm (**a**), simultaneous location in the focal plane of two (**b**) and three (**c**) focused vortices in accordance with their relative intensities. The dotted line in (**a**) shows the attenuation of the 613 nm intensity at which all three vortices can be successfully resolved in any combination with a sufficient contrast.

Simulation of a vortex with TC *m* = −2 leads to similar results (Figure 5).

Since the numerical aperture for this TC is higher, the focal spots become narrower, and the peak for *m* = −2, λ = 703 nm falls on the second ring from the focal spot with λ = 523 nm. Thus, the central peak is greater than for the *m* = −1. However, if only two extreme wavelengths are used, then the vortex with λ = 523 nm is seen from Figure 5a,b to be resolved with *p* = 7.7, and the vortex with λ = 703 nm—with *p* = 4. Even if the vortex with λ = 613 nm is present, the maxima of the two neighboring wavelengths can be distinguished in the beam due to narrower focal spots. If we decrease the incident intensity of the vortex λ = 613 nm to a possible contrast *p* = 2.1 (dotted line in Figure 5a), then all three wavelengths with TC *m* = −2 will not interfere with each other.

**Figure 6:**
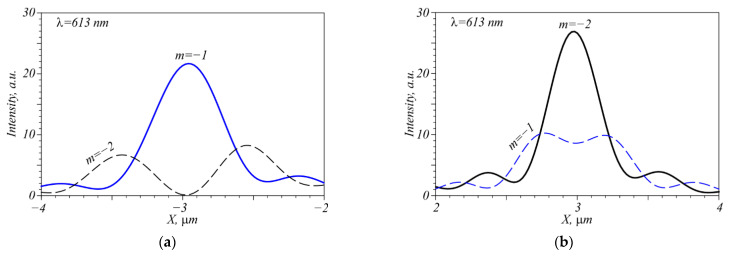
Intensity profiles through centers of the focal spots in the presence of two vortices with one wavelength λ = 613 nm and two TCs *m* = −1 and *m* = −2: −4 < x < −2 (**a**), 2 < x < 4 (**b**).

The question is how does the beam with TC *m* = −1 and any wavelength disturb the detection of individual wavelengths in the beam with TC *m* = −2? Side lobes of the vortex with TC *m* = −2 that can interfere with the focal spot of the vortex with TC *m* = −1 are shown in Figure 6a, the wavelength is 613 nm. Similar results for the focal spot of the vortex with TC *m* = −2 are shown in Figure 6b. The vortices are seen not to interfere with each other, however, at other wavelengths (523 nm, 703 nm), the peak shift does not allow them to be distinguished with the contrast *p* = 2 or higher. Therefore, at least two vortices with TC *m* = −1, *m* = −2 can be detected simultaneously on one wavelength, whereas for other wavelengths perhaps, time division multiplexing is necessary.

Note that vortices with larger TCs, *m* = −3 and **m* =* −4 were also examined. Metalenses for these TCs were found to be able to operate with an acceptable efficiency if they were located in the outer rings of the combined metalens. However, in this case the size of the simulation region grows significantly as well as the simulation time required to obtain dependences on the wavelengths. Therefore, these data are not presented in this article. Nevertheless, we find that this approach to the creation of the combined metalens is also acceptable both for larger values of the TC and for a bigger size of the metalens.

## 5. Conclusions

A combined metalens consisting of two separate metalenses capable of detecting separate vortices with different topological charges and different wavelengths is proposed. The metalens operates in a wide range of wavelengths from 500 to 700 nm, which makes it possible to determine the topological charge in the incident field and its wavelength. In addition, at one value of the topological charge, the shift of the focal spot along the transverse axis helps to simultaneously detect up to three vortices at different wavelengths with a sufficient contrast ratio (2 or more), which results in 8 different combinations of the presence or absence of vortices in the incident field to be distinguished. If two values of the topological charges are used, the number of possible combinations is greater. Such a metalens can be used in telecommunications for wavelength-division multiplexing and beam separation by the topological charge, which increases the amount of encoded information in the optical fiber.

## Data Availability

Not applicable.

## References

[1] Wang W., Guo Z., Zhou K., Sun Y., Shen F., Li Y., Qu S., Liu S. (2015). Polarization-independent longitudinal multi-focusing metalens. Opt. Express.

[2] Tian S., Guo H., Hu J., Zhuang S. (2019). Dielectric longitudinal bifocal metalens with adjustable intensity and high focusing efficiency. Opt. Express.

[3] Kim C., Kim S., Lee B. (2020). Doublet metalens design for high numerical aperture and simultaneous correction of chromatic and monochromatic aberrations. Opt. Express.

[4] Li M., Li S., Chin L., Yu Y., Tsai D., Chen R. (2020). Dual-layer achromatic metalens design with an effective Abbe number. Opt. Express.

[5] Hsiao H.-H., Chu C.H., Tsai D.P. (2017). Fundamentals and applications of metasurfaces. Small Methods.

[6] Shan D., Xu N., Gao J., Song N., Liu H., Tang Y., Feng X., Wang Y., Zhao Y., Chen X. (2022). Design of the all-silicon long-wavelength infrared achromatic metalens based on deep silicon etching. Opt. Express.

[7] Chantakit T., Schlickriede C., Sain B., Meyer F., Weiss T., Chattham N., Zentgraf T. (2020). All-dielectric silicon metalens for two-dimensional particle manipulation in optical tweezers. Photon. Res..

[8] Fan C., Chuang T., Wu K., Su G. (2020). Electrically modulated varifocal metalens combined with twisted nematic liquid crystals. Opt. Express.

[9] Ma X., He W., Xin L., Yang Z., Liu Z. (2022). Imaging performance of a mid-infrared metalens with a machining error. Appl. Opt..

[10] Qian Z., Tian S., Zhou W., Wang J., Guo H. (2022). Broadband achromatic longitudinal bifocal metalens in the visible range based on a single nanofin unit cell. Opt. Express.

[11] Xie Y., Zhang J., Wang S., Liu D., Wu X. (2022). Broadband polarization-insensitive metalens integrated with a charge-coupled device in the short-wave near-infrared range. Opt. Express.

[12] Hsiao H.-H., Chen Y.H., Lin R.J., Wu P.C., Wang S., Chen B.H., Tsai D.P. (2018). Integrated resonant unit of metasurfaces for broadband efficiency and phase manipulation. Adv. Opt. Mater..

[13] Liu M., Cao J., Xu N., Wang B. (2021). Broadband achromatic metalens for linearly polarized light from 450 to 800 nm. Appl. Opt..

[14] Wang W., Guo Z., Li R., Zhang J., Liu Y., Wang X., Qu S. (2015). Ultra-thin, planar, broadband, dual-polarity plasmonic metalens. Photon. Res..

[15] Ye H., Sun Q., Guo Z., Hou Y., Wen F., Yuan D., Qin F., Zhou G. (2021). Theoretical realization of single-mode fiber integrated metalens for beam collimating. Opt. Express.

[16] Wang G., Habib U., Yan Z., Gomes N., Sui Q., Wang J., Zhang L., Wang C. (2018). Highly efficient optical beam steering using an in-fiber diffraction grating for full duplex indoor optical wireless communication. J. Lightwave Technol..

[17] Shen Z., Xiang Z., Wang Z., Shen Y., Zhang B. (2021). Optical spanner for nanoparticle rotation with focused optical vortex generated through a Pancharatnam–Berry phase metalens. Appl. Opt..

[18] Guo Y., Zhang S., Luo X. (2021). Spin-decoupled metasurface for simultaneous detection of spin and orbital angular momenta via momentum transformation. Light Sci. Appl..

[19] Jin Z., Janoschka D., Deng J., Ge L., Dreher P., Frank B., Hu G., Ni J., Yang Y., Li J. (2021). Phyllotaxis-inspired nanosieves with multiplexed orbital angular momentum. eLight.

[20] Kotlyar V.V., Stafeev S.S., Nalimov A.G., O’Faolain L., Kotlyar M.V. (2021). A dual-functionality metalens to shape a circularly polarized optical vortex or a second-order cylindrical vector beam. Phot. Nanostr. Fund. Appl..

[21] Zeng J., Li L., Yang X., Gao J. (2016). Generating and separating twisted light by gradient–rotation split-ring antenna metasurfaces. Nano Lett..

[22] Mehmood M.Q., Mei S., Hussain S., Huang K., Siew S.Y., Zhang L., Zhang T., Ling X., Liu H., Teng J. (2016). Visible-frequency metasurface for structuring and spatially multiplexing optical vortices. Adv. Mater..

[23] Kotlyar V.V., Nalimov A.G., Stafeev S.S., Hu C., O’Faolain L., Kotlyar M.V., Gibson D., Song S. (2017). Thin high numerical aperture metalens. Opt. Express.

[24] Heckenberg N.R., McDuff R., Smith C.P., White A.G. (1992). Generation of optical singularities by computer-generated holograms. Opt. Lett..

[25] Lalanne P., Lemercier-Lalanne D. (1996). On the effective medium theory of subwavelength periodic structures. J. Mod. Opt..

[26] Kotlyar V.V., Nalimov A.G. (2017). A vector optical vortex generated and focused using a metalens. Comput. Opt..

[27] Stafeev S.S., O’Faolain L., Kotlyar V.V., Nalimov A.G. (2015). Tight focus of light using micropolarizer and microlens. Appl. Opt..

